# Electrochemical biosensors based on in situ grown carbon nanotubes on gold microelectrode array fabricated on glass substrate for glucose determination

**DOI:** 10.1007/s00604-022-05626-6

**Published:** 2023-01-16

**Authors:** Ankit Kumar Singh, Nandita Jaiswal, Ida Tiwari, Muhammad Ahmad, S. Ravi P. Silva

**Affiliations:** 1 Department of Chemistry (Centre of Advanced Study), Institute of Science, Banaras Hindu University, Varanasi, 221005 India; 2 Advanced Technology Institute, University of Surrey, Guildford, GU2 7XH Surrey UK

**Keywords:** Biosensor, Microelectrode arrays, Carbon nanotubes, Impedimetric, Glucose

## Abstract

A highly sensitive electrochemical sensor is reported for glucose detection using carbon nanotubes grown in situ at low temperatures on photolithographically defined gold microelectrode arrays printed on a glass substrate (CNTs/Au MEA). One of the main advantages of the present design is its potential to monitor 64 samples individually for the detection of glucose. The selectivity of the fabricated MEA towards glucose detection is achieved via modification of CNTs/Au MEA by immobilizing glucose oxidase (GO_x_) enzyme in the matrix of poly (paraphenylenediamine) (GO_x_/poly (p-PDA)/CNTs/Au MEA). The electrocatalytic and electrochemical responses of the proposed sensing platform towards glucose determination were examined via cyclic voltammetry and electrochemical impedance spectroscopy. The developed impedimetric biosensor exhibits a good linear response towards glucose detection, i.e., 0.2–27.5 µM concentration range with sensitivity and detection limits of 168.03 kΩ^−1^ M^−1^ and 0.2 ± 0.0014 μM, respectively. The proposed glucose biosensor shows excellent reproducibility, good anti-interference property, and was successfully tested in blood serum samples. Further, the applicability of the proposed sensor was successfully validated through HPLC. These results supported the viability of using such devices for the simultaneous detection of multiple electroactive biomolecules of physiological relevance.

**Graphical Abstract:**

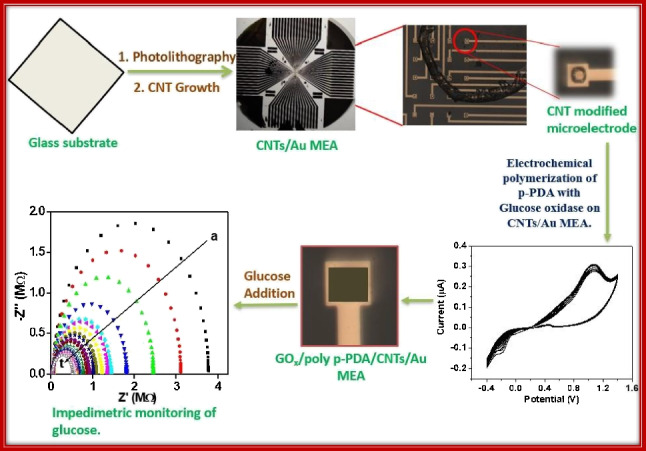

**Supplementary Information:**

The online version contains supplementary material available at 10.1007/s00604-022-05626-6.

## Introduction

Ever growing health issues demand accurate, rapid, and high-sensitivity testing and diagnostic systems to tackle various health conditions. Amongst many, diabetes mellitus is a well-known disease for its impact on public health globally. This is a metabolic disease which results from the inability of the body to produce enough insulin to cut down hyperglycemia [1]. A recent report published by WHO in 2016 indicates the rapid increase in the incidences of diabetes in past few years. Thus, sensitive, and reliable glucose monitoring in biological samples is crucial for the early detection and treatment of potential complications such as heart disease, renal failure, and blindness. Several techniques have been developed to measure glucose concentration based on various methods such as spectrophotometry [2], infrared spectroscopy [3], and colorimetry [4]. However, most of these methods are time consuming, require professional handling and expensive equipment, so are limited to clinical applications.

Electrochemical technique is a viable method for the frequent determination of glucose level due to its significant advantages of speed, robustness, and simplicity. Various electrochemical enzymatic glucose biosensors work on the principle of immobilization in which glucose oxidase (GO_x_) enzyme get immobilized using different functional nanomaterials [5]. The most common electrochemical method used in glucose sensors is probably based on amperometry. But in recent years, the use of EIS as a transduction mechanism in these sensors has been proposed. EIS techniques are more useful to study the analyte in lower concentrations, the electrode/electrolyte interface, and the kinetics of the electrode surface. The use of EIS biosensors has expanded dramatically as a result of its easy manipulation, quick reaction, ability to be miniaturized, and readiness for lab-on-a-chip integration with affordable and real-time monitoring to detect extremely low concentrations [6].

Microelectrode arrays (MEAs) have been used for correlating biological activities with electrical signals [7] and measuring the electrochemical response from biomolecules or organic analytes [8]. The MEA can be used to sense analytes present in a solution with the possibility of designing highly sensitive and reliable biosensors. The microelectrodes have a number of advantages over conventionally sized macroelectrodes (> 1 mm), including high mass transport due to radial diffusion, rapid attainment of steady-state, low ohmic drop, reduced non-faradaic charging current, independence from convection, and the ability to increase current responses with MEAs [9]. Photolithography, widely used in the semiconductor industry, is utilized in the fabrication of electrochemical sensors at the micro scale. The photolithographic technique is highly precise and allows the designing of microscale electrode arrays [8]. Furthermore, the sensitivity and specificity of each microelectrode for individual biological molecules or chemical analytes can be tuned and electrically multiplexed for precise reporting to achieve highly sensitive biosensors.

Carbon nanotubes are extensively used in electrochemical testing to enhance the electrical signal. CNTs exhibit a number of attractive properties including the potential for modification, biocompatibility, high electrical conductance, and high-aspect ratio which produces an increased surface area and improved charge transfer rate [10]. The in situ growth of CNTs directly on electrodes is highly desirable in order to exploit the intrinsic properties of CNTs. CNT growth is generally achieved at elevated temperatures, (> 700 °C), and hence, unsuitable for substrates that are temperature sensitive such as glass [10]. We use a photothermal CVD technique for achieving the growth of CNTs at lower substrate temperatures (< 450 °C), which allows us to conduct the growth on a glass substrate; further details about the technique can be found elsewhere [11]. Combining the advantages of MEA, photolithography, and carbon nanotubes, CNTs/Au MEA can be designed having 64 microelectrodes. The use of CNTs/Au MEA produced a significant increase in the current and high enzyme loading capacity owing to the enhanced surface area in the presence of CNTs. This high-performance sensing platform can, not only be used for glucose detection in multiple samples, but these 64 electrodes can be used individually for the detection of other useful analytes present in blood samples by using a specific recognition element on each microelectrode intimately integrated to an electrochemical transduction system beyond the stage of research for point-of-care medical applications. Our collaborative research team is also working on some other prototypes of CNTs/Au MEA-based electrochemical sensors and biosensors that may find practical applications in the near future. The findings of these results will be communicated separately shortly and beyond scope of the present paper.

The application of immobilized biochemical compound such as enzymes on microelectrodes or the electrodes modified with nanocomposite materials has the potential for the development of miniaturized biosensors. However, the immobilization of different enzymes on a selected microelectrode is challenging. Several traditional methods such as chemical cross-linking, adsorption, and entrapment are used to immobilize the enzymes, but these methods do not show specificity and selectivity in the deposition of specific enzymes onto a desired microelectrode of an array [12]. Electrodeposition, however, is an excellent method to achieve specificity and selectivity of enzyme onto a particular electrode [13]. Poly (paraphenylenediamine) electrosynthesized from the p-PDA monomer have found widespread use as a permselectivity barrier in the biosensor. The incorporations of a permselective layer in the development of biosensor further minimize the interference problem [14]. Moreover, immobilization of glucose oxidase (GO_x_) enzyme in the poly (p-PDA) matrix helps in achieving the excellent selectivity of the fabricated GO_x_/poly (p-PDA)/CNTs/Au MEA.

Herein, we describe the fabrication of a highly sensitive, photolithographically defined CNTs/Au MEA based 3rd generation electrochemical enzymatic glucose biosensors on a glass substrate where direct transfer of electron occurs between the surface of the electrode and enzyme’s redox-active site without any redox mediator. The absence of a mediator increases the selectivity owing to the low operating potential. The glass substrate is preferred over Si because of its low cost and easy availability all over the world. Direct CNTs growth on glass substrates is challenging in conventional techniques because of the high-temperature involvement (700–1100 °C); however, we have demonstrated the growth on glass substrate using our photo-thermal CVD (PTCVD), where the substrate temperature is kept below 400 °C [11]. The developed GO_x_/poly (p-PDA)/CNTs/Au MEA-based electrochemical glucose biosensing platform is simple, small-sized, biocompatible, cost efficient, easy to use, and requires a small volume of sample. Further, it was potentially applied for measuring the level of glucose in human blood serum sample with excellent recovery to evaluate its real sample application.

## Experimental

### Chemicals and apparatus

Glucose oxidase (GO_x_) (from *Aspergillusniger* 20,000 units/g, EC 1.1.3.4), β-D-Glucose, and p-phenylenediamine (p-PDA) were purchased from Sigma-Aldrich, India. Triple-distilled water was used for the preparation of all the solutions. Phosphate buffer solutions (PBS) containing 0.1 M Na_2_HPO_4_/NaH_2_PO_4_ were used and the pH was maintained with diluted solutions of NaOH or H_3_PO_4_. The 10 mM pre-polymerization mixture of p-PDA was made in PBS 7.0. For the preparation of glucose stock solution, PBS (pH 6.5) was used. It was then kept at 4 °C overnight for achieving anomeric equilibrium.

The EML photoresist spinner controller EMS 6000, precision hot plate, and Suss Microtec MA1006 Mask Aligner were used in photolithographic patterning. JLS loadlocked sputter coater and Univex ebeam evaporator were used for metal deposition on the substrate and growth of CNTs was performed using Surrey Nano Systems SNS1000n photothermal chemical vapor deposition (PTCVD). Scanning electron microscopic (SEM) measurements were conducted using Tescan MIRA II SEM Field Emission (InBeam, 30 kV accelerating voltage), and Raman spectra were recorded on XploRa Plus Multiline Confocal Raman Microscope (532 nm laser, 2400 grating, 100 × objective lens, 10% filter). Zeiss Evo 18 Research scanning electron microscope was used for energy-dispersive X-ray (EDX) spectroscopy. High performance liquid chromatography (HPLC) analysis was performed on Binary HPLC (Model-1525), Waters Corporations. Absorbance was measured on a Hitachi U3900 spectrophotometer. Metrohm electrochemical Potentiostat/Galvanostat (Nova 1.8 software) is used for electrochemical studies using a working electrode (CNTs/Au MEA), counter electrode (platinum wire), and reference electrode (Ag/AgCl).

### MEA fabrication and CNT growth

**Fig. 1:**
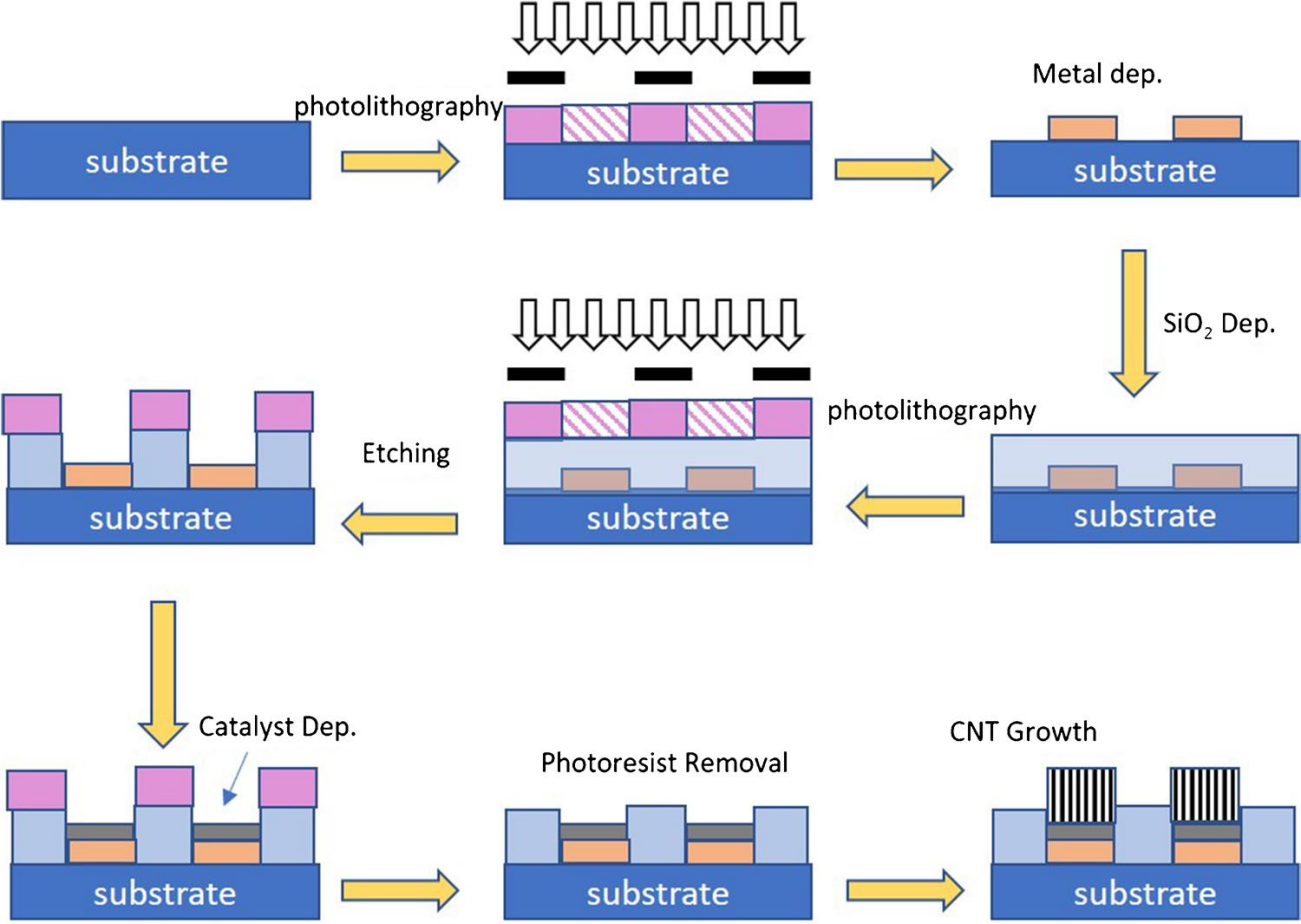
Process flow for fabrication of CNTs/Au MEA as described [11]

The fabrication of MEA (c.f. Figure 1) on a glass substrate was carried in such a way that several microelectrodes are arranged in a defined pattern of 8 × 8 to form a matrix of 64 microelectrodes [8]. The dimension of each microelectrode is about 10 μm × 10 μm while one electrode in each corner is about 100 μm × 100 μm in dimension. Prior to the patterning, the glass substrate was carefully cleaned for the microfabrication process through successive sonication in acetone, isopropanol, and methanol respectively. Further, it was ashed under oxygen plasma for 5 min at 100 W rf power. After the cleaning, the substrate was patterned photolithographically using the first mask to define microelectrodes, connecting lines, and contact pads. Ti (4 nm) was DC sputtered on the patterned sample and then Au (95 nm) was evaporated on it. The sample was then immersed in NMP-1165 solvent for lift-off. After this, SiO_2_ (230 nm) was evaporated on the sample, followed by photolithographic patterning using the second mask. The areas defined in the second patterning step are processed for reactive ion etching using a mixture of gas containing CF_4_ (30 sccm) and Ar (10 sccm) at 60 mT with 100 W power to access electrical connections and contact pads for electrode-analytes contact. After etching, Al (8 nm) and Fe (2 nm) were DC sputtered as a catalyst for CNT growth, and the photoresist was removed using NMP-1165 solvent. Finally, CNTs are grown on MEAs at low substrate temperature (< 400 °C) using PTCVD; details about PTCVD can be found in our previous publication [11, 15]. Briefly, the PTCVD utilizes a water-cooled substrate table to keep the substrate temperature low and an array of optical lamps configured to deliver optical energy directly to the catalyst. The CNT growth process involves pre-heating the sample under the environment of H_2_ (10 min) followed by the CNT growth using a C_2_H_2_/H_2_ (10/100 sccm) mixture at 2 Torr pressure. Apart from the MEA sample, growth of CNTs using the same conditions is also performed on a 1 × 1 cm^2^ plane Si substrate where 50 nm TiN, 10 nm Al, and 3 nm Fe was sputtered to act as catalyst and catalyst support layers. This sample will be referred as CNTs/Si hereafter in the manuscript.

### Glucose biosensor fabrication

Glucose analysis was performed by immobilizing the GO_x_ enzyme on the CNTs grown over the electrode surface. The immobilization of glucose oxidase can be done by using a polymer matrix and here, p-PDA was electropolymerized on the microelectrode surface of the array for the formation of a polymer matrix that encapsulates the GO_x_ enzyme. The immobilization of the GO_x_ enzyme in the p-PDA polymer matrix might help in improving the storage properties of enzyme, its handling and stability [12].

#### Electropolymerization of p-PDA

The electropolymerization of p-PDA was carried in between − 0.4 to 1.2 V vs. Ag/AgCl at microelectrodes of the array using 10 mM p-PDA in 0.1 M PBS buffer of pH 7.0, using the cyclic voltammetric technique for 20 cycles at sweep potential of 50 mV/s. The electropolymerization of p-PDA was firstly studied on 1 × 1 cm^2^ CNTs/Si electrode and then on microelectrodes of CNTs/Au MEA [16].

#### Glucose oxidase immobilized poly (p-PDA)

To fabricate enzyme electrode viz. GO_x_/poly (p-PDA), 1 mg of GO_x_ was added to the deaerated 0.1 M PBS of pH 7.0 having 10 mM p-PDA. The electropolymerization was performed by applying the potential from − 0.4 to 1.2 V vs. Ag/AgCl electrode at a sweep potential of 50 mV/s. The same method is followed for CNTs/Si wafer and then on microelectrodes of CNTs/Au MEA to produce GO_x_/poly (p-PDA)/CNTs/Si wafer and GO_x_/poly (p-PDA)/CNTs/Au MEA, respectively [16].

### Determination of enzyme activity

The conventional colorimetric technique described elsewhere was used to measure the activity of free GO_x_ enzyme by analyzing the amount of H_2_O_2_ produced during the catalytic reaction of β-D-glucose and GO_x_ [17]. The specific activity of the free GO_x_ was found to be close to the expected value of 20,000 units/g. However, it is critical to test the activity of the immobilized enzyme and to determine the ideal concentration of active sites available even after immobilization. It is because the conventional biochemical methods cannot be accurately assayed for measuring the activity of immobilized enzyme owing to the presence of solid support [18]. Thus, because of these difficulties, we were unable to check the activity of the GO_x_ enzyme after electrodeposition. Moreover, the method used in this study for immobilization of GO_x_ enzyme involves co-deposition on the electrode surface where GO_x_ enzyme was entrapped in the polymer matrix of p-PDA. Hence, it is expected that the activity of the immobilized enzymes would be maintained or there should be negligible loss in activity after immobilization in the polymer matrix [18].

### Electrochemical measurements

The fabricated GO_x_/poly (p-PDA)/CNTs/Si wafer and GO_x_/poly (p-PDA)/CNTs/Au MEA were initially employed for electrochemical determination of glucose through CV in the presence of 2-mL PBS electrolyte (0.1 M, pH 6.5). The potential was varied at the scan rate of 20 mVs^−1^. In a typical measurement, GO_x_/poly (p-PDA)/CNTs/Si wafer and one microelectrode (out of 64) of the GO_x_/poly (p-PDA)/CNTs/Au MEA were subjected to − 0.8 V to 0.9 V and − 0.5 to 0.9 V potential ranges, respectively at the scan rate of 20 mVs^−1^. The cyclic potential was swept until stable voltammograms were obtained. Then, various glucose concentrations were added to the supporting electrolyte and stirred sufficiently. Their corresponding responses were measured through CV in the same potential ranges on both working electrodes. Further, EIS measurements were also performed in the absence and presence of various glucose concentrations to assess the impedimetric response at microelectrode of the fabricated GO_x_/poly (p-PDA)/CNTs/Au MEA as an enzymatic electrochemical glucose biosensor. The oscillation frequency was applied in the range of 10 kHz to 0.1 Hz and the set potential was applied to be − 0.30 V vs Ag/AgCl at which best catalysis was observed on GO_x_/poly (p-PDA)/CNTs/Au MEA. EIS was further performed to check the reproducibility and stability of the developed sensing platform as well as applicability in the real samples. Each analytical studies were repeated three times under the similar laboratory conditions.

## Results and discussion

### Physical characterizations

**Fig. 2:**
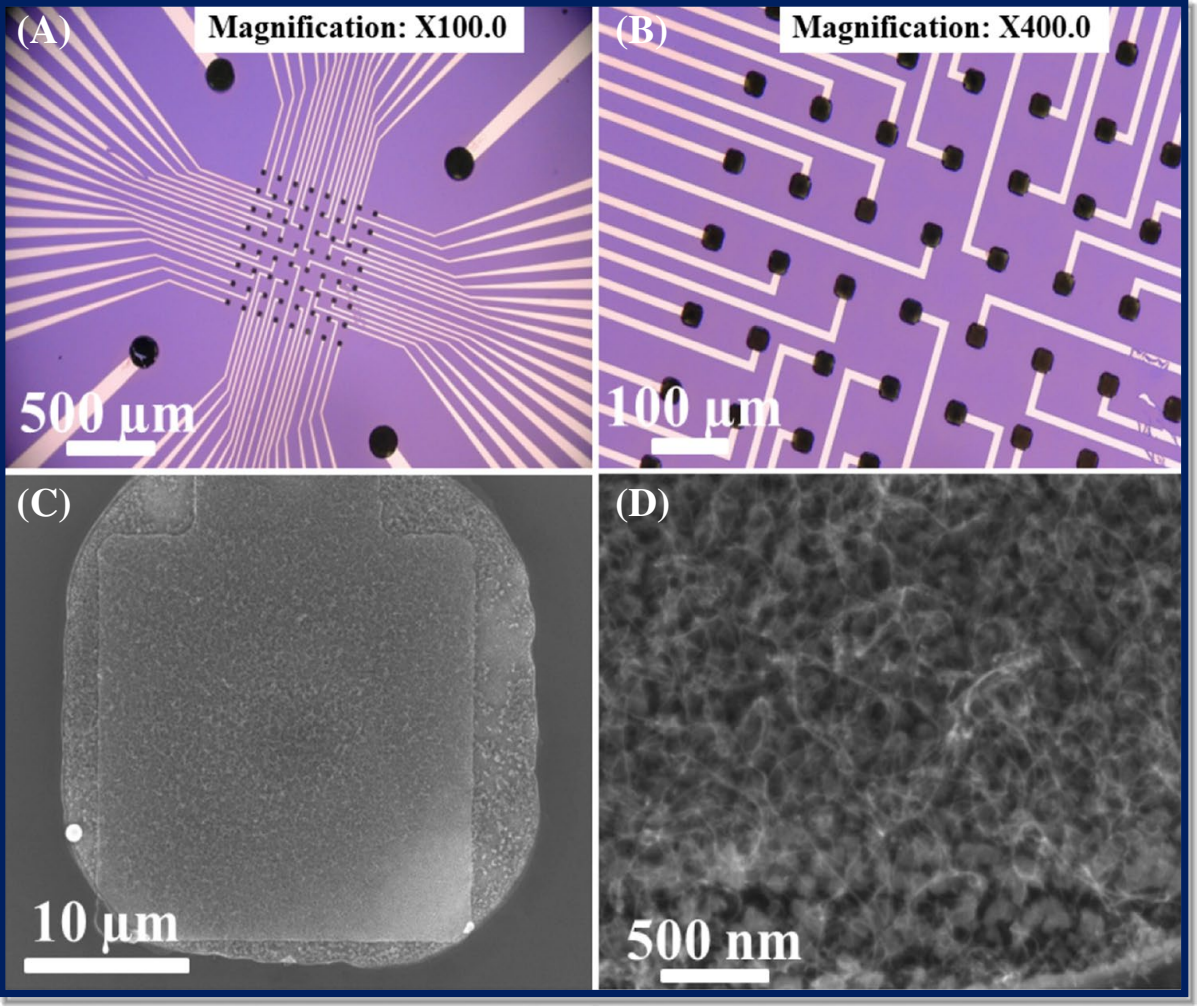
Optical images showing fabricated Au MEAs with microelectrodes, connecting lines, and contact pads (**A** and **B**). SEM images of CNTs grown on microelectrode (**C** and **D**)

The fabricated CNTs/Au MEAs and CNTs/Si samples were characterized by optical microscopy, SEM, and Raman spectroscopy. The optical images of the fabricated Au MEAs with grown CNTs showing microelectrodes in the center, connecting lines and contact pads are represented in Fig. 2A and B. The high magnification SEM image of CNTs at the center of microelectrodes for the CNTs/Au MEA sample is demonstrated in Fig. 2C and D, whereas the image for CNTs/Si sample is given in Fig. S1 in supplementary information. The images in Fig. 2C and D show short (~ 1 µm in length) and tangled CNTs grown uniformly over the whole microelectrode surface, whereas the CNTs on CNT/Si sample are vertically aligned with their length around 2 µm [19]. The EDX report represented in Fig. S2A confirms the presence of an Au metal layer under CNTs. It should be noted that some parasitic CNT growth occurred in areas around the microelectrode as can be seen in Fig. 2C. This happened due to the isotropic etching of the oxide layer, resulting in the catalyst deposition around the electrode and subsequent growth of CNTs. However, these CNT are not taking part in the conduction or detection process since these are not connected with the bottom metal (Au) layer. Moreover, the SEM image of one microelectrode of GO_x_/poly (p-PDA)/CNTs/Au MEA is illustrated in Fig. S2B, where polymer gel can be observed over the surface of the microelectrode. The change in surface topography of microelectrode can be observed from Fig. 2D to Fig. S2B owing to the successful electropolymerization of poly (p-PDA) gel containing GO_x_ enzyme and this might result in rapid diffusion of glucose on the interface of modified MEA.

**Fig. 3:**
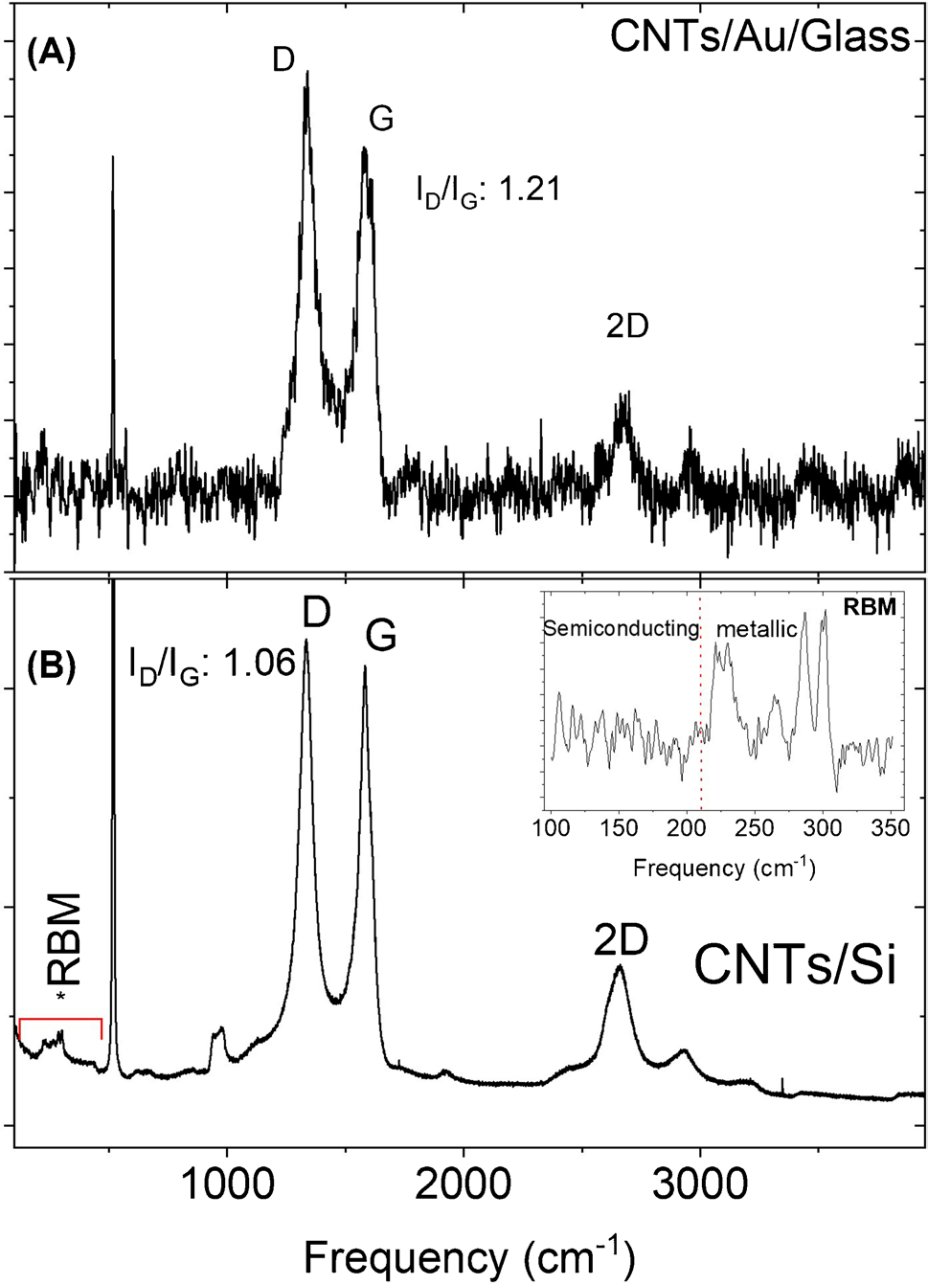
**A** Raman spectrum (taken using 532-nm laser wavelength) of the CNTs grown on Au MEAs, showing distinct characteristics of carbon nanotubes with the G-peak centered at 1583 cm^−1^, the D-Peak at 1340 cm^−1^, and the 2D peak at 2675 cm^−1^. **B** Raman spectrum of CNTs grown on Si sample. Inset shows magnified RBM region. All the peaks in the RBM region are found between 210 and 300 cm^−1^, which indicates the growth of metallic single-walled carbon nanotubes

The vibrational attributes of the CNTs grown on Si and glass substrates were investigated by Raman spectroscopy using a laser of wavelength 532 nm (Fig. 3). Distinct first and second-order Raman features are observed on both samples. The disorder-induced A_1g_ Raman mode in the form of D-band is observed at ~ 1340 cm^−1^ on both samples and the G-band which is due to the E_2g_ vibrations of the sp^2^ bonded carbon atoms (Raman active) is observed at 1578 cm^−1^ for CNTs/Au MEA sample (c.f*.* Figure 3A) and at 1590 cm^−1^ for CNTs/Si sample (c.f. Figure 3B). As, the G-band indicates graphitization in the material and the D-band is due to the structural defects, their intensity ratio (I_D_/I_G_) is commonly used to judge the structural quality of the carbonaceous materials; lower I_D_/I_G_ values indicate higher structural quality and vice versa. The I_D_/I_G_ value of 1.21 indicates relatively higher structural defects in our CNTs, which may be because of disorders in the hexagonal structure, broken bonds, and the presence of amorphous carbon. However, defective CNTs may be advantageous in this application as they may provide more attachment sites for enzymes and subsequent glucose detection. The 2D-band at 2675 cm^−1^ is the overtone of the D-band but independent of the defects [11]. The peaks observed between 100–350 cm^−1^ (shown in the inset of Fig. 3B) are due to the radial breathing mode (RBM) and indicate the presence of single-walled CNTs. The peaks between 150–210 cm^−1^ are attributed to the presence of semiconducting SWCNTs, and those between 210–300 cm^−1^ indicate metallic SWCNTs [11]. The observed peaks in the RBM region in Fig. 3B lie between 210 and 300 cm^−1^, indicating the growth of metallic CNTs, which is well suited for this application. Further characterization of PTCVD-grown CNTs at various conditions using TEM, SEM, Raman spectroscopy, EDX, and XPS can be found in our previous studies [11, 15].

### Electrochemical characterization of poly (p-PDA)

**Fig. 4:**
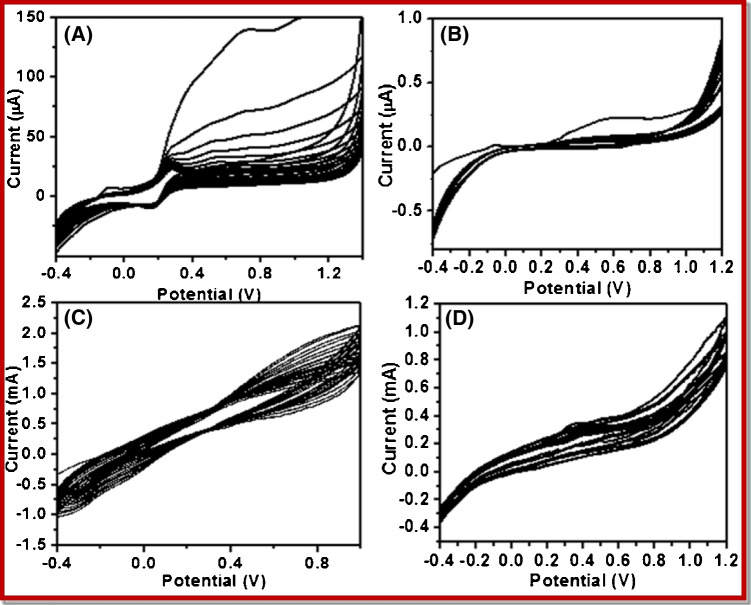
Electropolymerization of 10 mM solution of p-PDA at 50 mVs^−1^ for 10 cycles in PBS of pH 7.0 at **A** CNTs/Si wafer and **B** CNTs/Au MEA. Electropolymerization of 10 mM p-PDA with 1 mg/mL glucose oxidase for 20 cycles at 50 mVs^−1^ in PBS (pH = 7.0) at **C** CNTs/Si wafer and **D** CNTs/Au MEA

The oxidation of the poly (p-PDA) isomers at the CNT-grown silicon wafer (CNTs/Si wafer) and on the microelectrode of Au MEA is irreversible and can be seen in Fig. 4A and B, respectively. The oxidation peak potential was found to be 0.27 V for poly (p-PDA) from the first voltammetric scan cycle. The order of the oxidation is the same for both CNTs/Si wafer and microelectrode of CNTs/Au MEA, as can be seen in Fig. 4A and B. The electropolymerization current collapsed due to the self-sealing nature of poly (p-PDA) as it accumulated on the electrode surface and hence, a continuous decrease in peak current was seen due to the formation of a compact, insulating poly (p-PDA) film that causes hindrance on the surface of the electrode [14]. Further, the addition of GO_x_ on (p-PDA) solution has not shown any noticeable change in the voltammetric response. However, during the electropolymerization of p-PDA in the presence of glucose oxidase enzyme (c.f. Figure 4C and D), the current response was observed to be very high compared to the electropolymerization performed in the absence of glucose oxidase enzyme (c.f. Figure 4A and B). The increase in current with the addition of glucose oxidase may be attributed to the interaction between GOx enzyme and p-PDA that slows down the collapse in current and result in an increase in the rate of electrodeposition of poly (p-PDA) [14]. Further, the oxidation and reduction peak potentials remain unchanged even after repeated cycles indicating the stability of prepared electrodes.

### Optimization conditions for electropolymerization

**Fig. 5:**
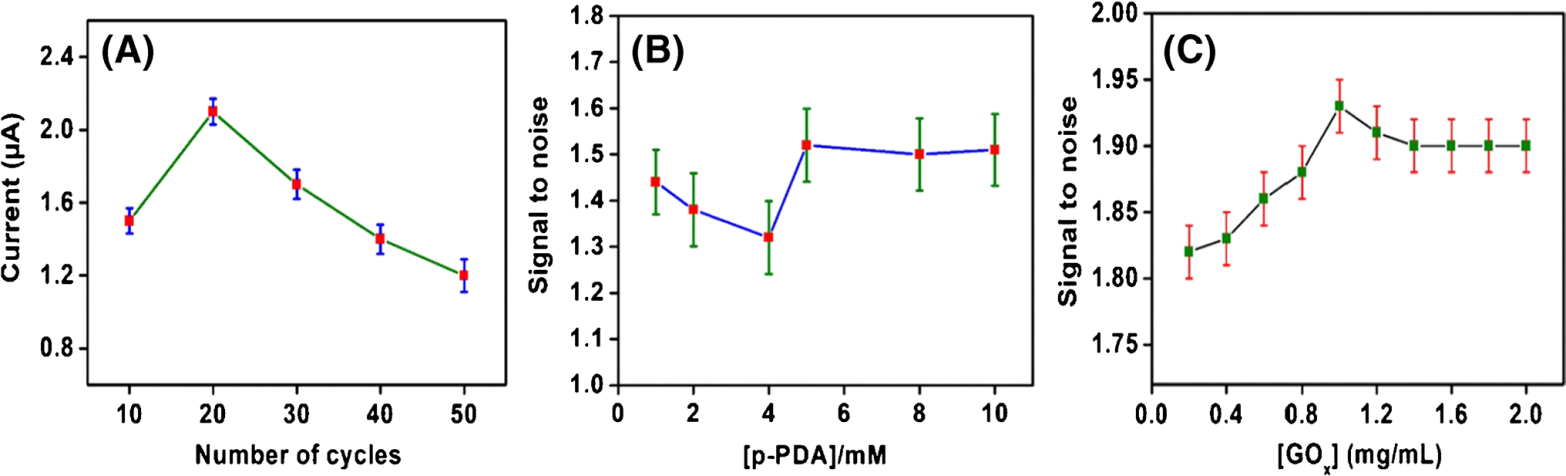
Results obtained for optimization of the electropolymerization process. **A** Number of CV cycles; **B** (p-PDA) concentration, and **C** (GO_x_) concentration

The process of electropolymerization was optimized to get maximum response for the redox probe. The number of CV cycles, concentration of p-PDA monomer, and concentration of GOx enzyme were optimized. The variation in the current response with different optimization parameters is represented in Fig. 5.

It is possible to adjust the thickness of the polymer by controlling the number of potential cycles during electropolymerization. The formation of a thick (non-conductive) polymer obstructs electron transport and/or diffusion of the redox probe to the electrode surface that might result in a lowering in current values [20]. As such, the present study has concentrated on the development of a thin film biosensor. For this, the conditions were optimized for the formation of a thin film of the same thickness. For optimization, polymerization cycles were varied from 10 to 50 at one microelectrode (out of 64) of CNTs/Au MEA using the same sweep potential as shown in Fig. 5A. It was observed that maximum current was obtained when polymerization was performed for 20 cycles at sweep potential of 50 mV/s. The thickness of the film after 20 CV cycles of electrodeposition was calculated using relation [21];

$$e=\frac{M \times Q}{n \times F \times A\times \rho }$$

where *e* represents the thickness of the electrodeposited film, *M* denotes the molar mass of the polymer (108.14 g mol^−1^), *n* represents the number of electrons transferred (*n* = 1), *F* is the Faraday constant (96,485 C.mol^−1^), *A* is the area of working electrode surface (1 × 10^−6^ cm^2^), and *ρ* signifies the density of the polymer (*ρ* = 1). *Q* is the electrical charge consumed during the polymerization process and its value for 20 CV cycles of electropolymerization was calculated from the area under CV curves and it was found to be 0.0072 µC. The thickness of the electrodeposited polymer on one microelectrode of CNTs/Au MEA was found to be 80.70 nm. Moreover, when we performed the electropolymerization study for 20 cycles on further days for each experiment in the same buffer solution, in the same potential range, and in the same experimental condition, we found that the current was reproducible which indicates the reproducibility in thickness of the films formed.

The optimal concentration of p-PDA monomer required for the electropolymerization process was tested in the presence of 1.0, 2.0, 4.0, 5.0, 8.0, and 10 mM as represented in Fig. 5B. It was observed that the current response decreases continuously upto 4.0 mM and then increases. However, only a slight increase in current response was observed when p-PDA concentration was chosen between 5.0 mM to 10 mM, and hence, a 10 mM concentration of p-PDA was taken for the electropolymerization process.

Furthermore, the signal produced is proportional to the quantity of enzyme that binds and it affects the sensitivity of the biosensor. If there is not enough enzyme, the current signal may be extremely low. The signal-to-noise ratio suffers and it becomes difficult to identify the difference between various concentration standards if the background is too high. Hence, different concentration of GO_x_ enzyme between 0.2 and 2.0 mg/mL was also tested as illustrated in Fig. 5C and the optimal concentration of GO_x_ was found to be 1 mg/mL. Initially, there was an increase in current response with an increase in the concentration of GO_x_ between 0.2 and 1.0 mg/mL after which the current response slightly decreases and becomes constant owing to the saturation of enzyme loading capacity of the polymer matrix.

Thus, after considering the optimal values of all the optimization parameters, the electropolymerization of 10 mM p-PDA solution containing 1 mg/mL GO_x_ enzyme was allowed for 20 CV cycles of electrodeposition process on one microelectrode at a time out of 64 of the fabricated CNTs/Au MEA. The thickness of GO_x_ immobilized poly (p-PDA) film on the microelectrode of the CNTs/Au MEA was found as 85.18 nm and the value of electrical charge consumed during the respective polymerization process was 0.0076 µC. It was observed that the electrical charge and thickness of poly (p-PDA) in the presence of GO_x_ enzyme slightly greater than in the absence of GO_x_ that might be due to the interaction of p-PDA with the GO_x_ enzyme. Further, the electropolymerization was performed in the presence of PBS containing GO_x_ and p-PDA and thus, it can be said that GO_x_ and p-PDA were co deposited to the microelectrode surface. The GO_x_ enzyme was entrapped in the polymer matrix of poly (p-PDA). During the entrapment of GO_x_ in poly (p-PDA), there is no possibility for the formation of a covalent linkage between GO_x_ and poly (p-PDA) and no structural distortions [22].

### Electrochemical response of glucose at different electrodes

**Fig. 6:**
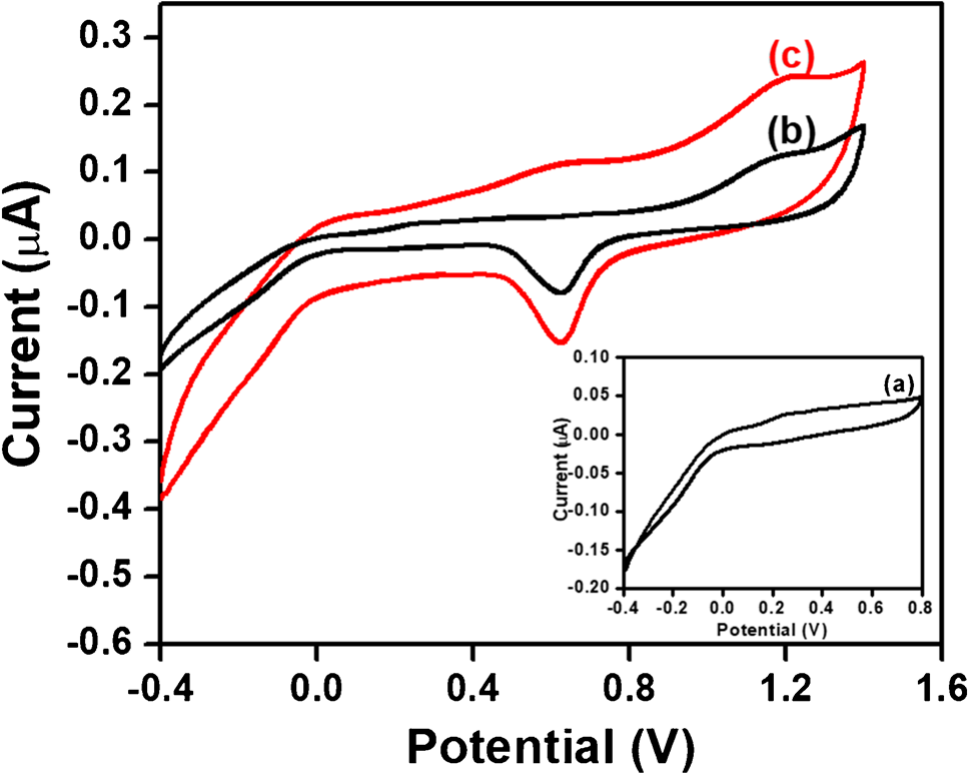
Cyclic voltammograms of 2.5 mM glucose in PBS (pH 6.5) at one microelectrode (out of 64) of CNTs/Au MEA **a** poly (p-PDA)/CNTs/Au MEA **b** and GO_x_/poly (p-PDA)/CNTs/Au MEA **c** at scan rate of 0.02 Vs.^−1^

CV was recorded on different modified and unmodified electrodes in the presence of glucose to compare their electrochemical performances. Figure 6 represents the CVs of one microelectrode (out of 64) of CNTs/Au MEA (curve a), poly (p-PDA)/CNTs/Au MEA (curve b), and GO_x_/poly (p-PDA)/CNTs/Au MEA (curve c) in the presence of 2.5 mM glucose in PBS (pH 6.5) at a scan rate of 0.02 Vs^−1^. It was observed that there is no oxidation peak for glucose on CNTs/Au MEA and on poly (p-PDA)/CNTs/Au MEA that suggests about the absence of glucose oxidation on these electrode surfaces. However, there is the presence of a well-defined oxidation peak of glucose at GO_x_/poly (p-PDA)/CNTs/Au MEA owing to the enzymatic oxidation of glucose in the presence of immobilized GO_x_ enzyme. Further, the current signal of GO_x_/poly (p-PDA)/CNTs/Au MEA is higher than that of poly (p-PDA)/CNTs/Au MEA, indicating the effective electron transfer ability of GOx towards the oxidation of glucose. This might be because of the electrocatalytic activity of GO_x_, poly (p-PDA), and CNTs/Au MEA as their synergistic effect towards the oxidation of glucose.

### Cyclic voltammetry response of glucose on GO_x_/poly (p-PDA)/CNTs/Si wafer and GO_x_/poly (p-PDA)/CNTs/Au MEA

**Fig. 7:**
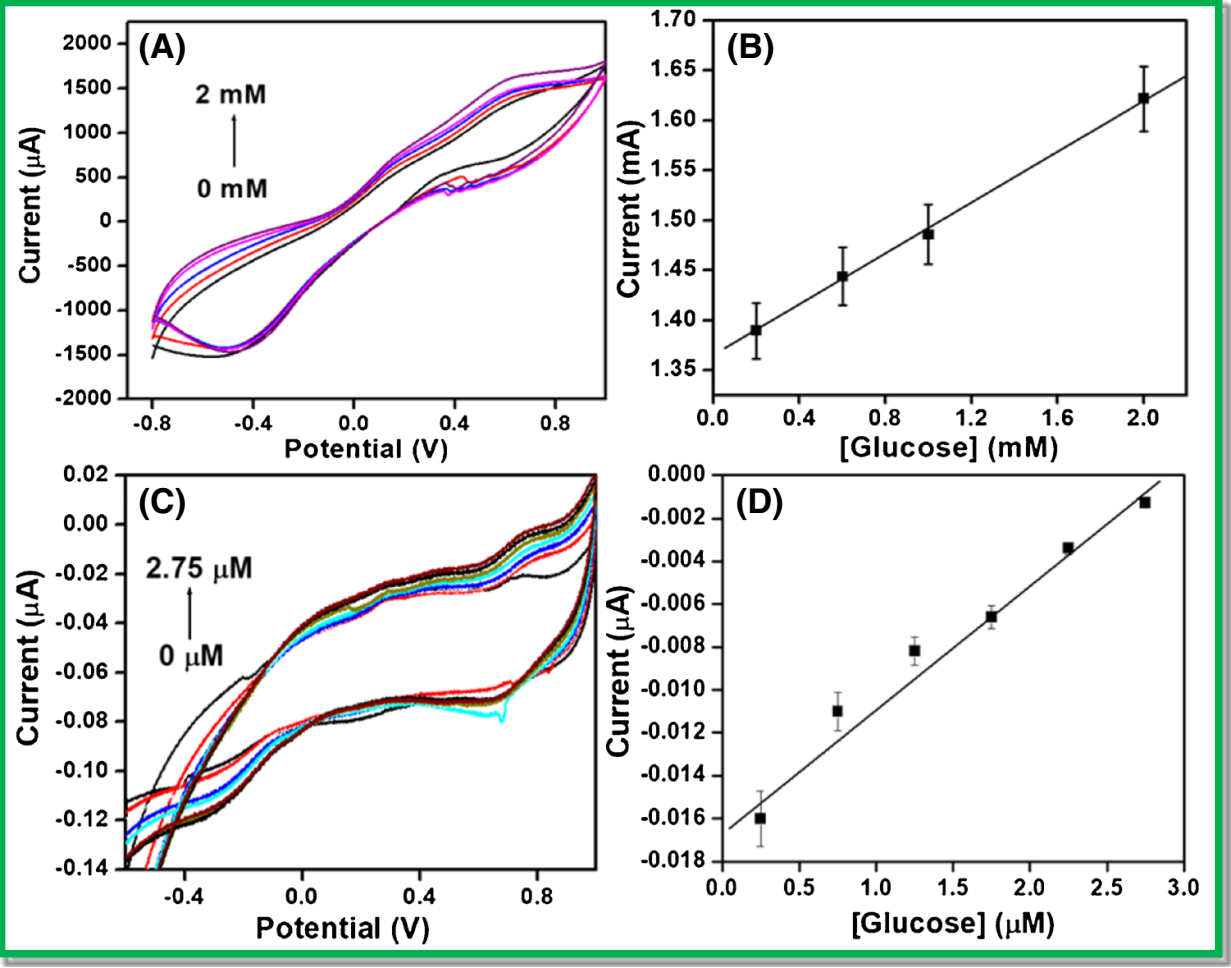
Cyclic voltammograms for different additions of glucose at 20 mVs^−1^ on **A** GO_x_/poly (p-PDA)/CNTs/Si wafer and **C** GO_x_/poly (p-PDA)/CNTs/Au MEA in PBS of pH 6.5. The corresponding calibration plot for **B** GO_x_/poly (p-PDA)/CNTs/Si and **D** GO_x_/poly (p-PDA)/CNTs/Au MEA

The influence of the concentration of glucose was determined by the addition of different glucose concentrations on GO_x_/poly (p-PDA)/CNTs/Si wafer and on one microelectrode (out of 64) of GO_x_/poly (p-PDA)/CNTs/Au MEA in PBS electrolyte of pH 6.5 at 20 mVs^−1^ scan rate. Figure 7 A and C shows the CV response for various glucose concentrations added, from 0 to 2 mM and 0 to 2.75 µM at GO_x_/poly (p-PDA)/CNTs/Si wafer and GO_x_/poly (p-PDA)/CNTs/Au MEA, respectively. The corresponding calibration plot for the determination of glucose at GO_x_/poly (p-PDA)/CNTs/Si wafer and GO_x_/poly (p-PDA)/CNTs/Au MEA are represented in Fig. 7B and D, respectively. The anodic peak potential was observed at 0.60 V signifying the oxidation of glucose to gluconic acid which is catalyzed by GO_x_ molecules through direct electron transfer. The reduced form of GO_x_ is oxidized into its native form at the electrode surface for ensuing the enzymatic step. While at negative potentials, there was a subsequent reduction of H_2_O_2_, and this process of H_2_O_2_ reduction might be due to the synergistic effect of poly (p-PDA)/CNTs on Si wafer and gold arrays as well. Glucose determination in this sensing device was done by monitoring the oxidation of glucose only in order to ensure specificity; as there is a possibility of interference from the reduction of other analytes which may be present in real samples beyond the negative potentials of − 0.2 V observed for H_2_O_2_ [16]. Chronoamperometry and differential pulse voltammetry study were similarly conducted on the prepared electrodes but due to the small surface area of the prepared electrodes, accurate data could not be realized. To further explore the options in the development of a useful technology for electrochemical impedance studies, we conducted the very encouraging demonstration of the applicability of prepared electrodes for highly sensitive monitoring of glucose.

### Impedimetric glucose response study over GO_x_/poly (p-PDA)/CNTs/Au MEA

**Fig. 8:**
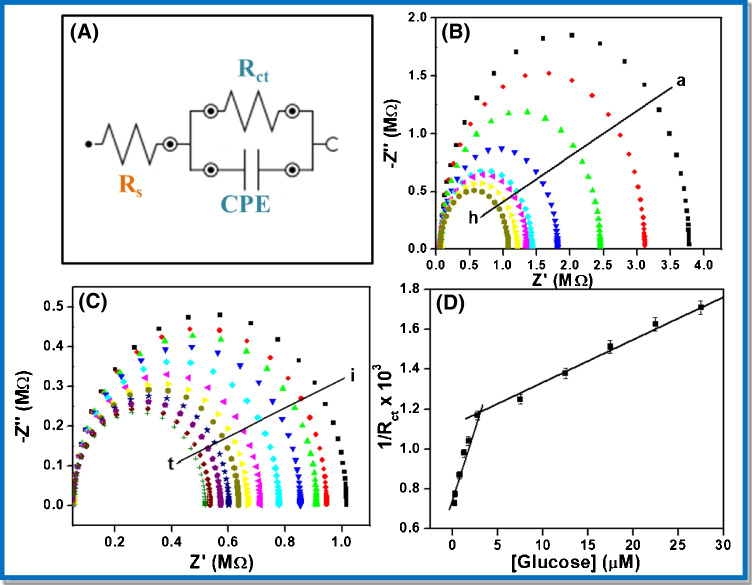
**A** Randles and Ershler model circuits used for EIS in frequency range of 0.1 to 10 kHz. **B** and **C** EIS response of glucose on one microelectrode (out of 64) of GOx/poly (p-PDA)/CNTs/Au MEA in PBS of pH 6.5 in the concentration range of 0 to 27.5 µM. **D** Corresponding calibration plot for glucose addition

For fast and sensitive response occurring on the electrode–electrolyte interface, the electrochemical impedance spectroscopy (EIS) is mostly used. It comprises of a semicircle section witnessed at higher frequencies that implies about limited electron transfer process with an addition to a linear part at the lower frequencies ascribed to a diffusion-limited electron transfer process. The diameter of the semicircle indicates the charge transfer resistance (R_ct_) value and the reduction in semicircle diameter implies a decrease in the R_ct_ value. From the experimental impedance spectra, numerical values of R_ct_ can be obtained by fitting the data onto an equivalent circuit built on a modified Randles and Ershler model, as shown in Fig. 8A. For evaluation of the analytical response at GO_x_/poly (p-PDA)/CNTs/Si and at one microelectrode (out of 64) of GO_x_/poly (p-PDA)/CNTs/Au MEA as an enzymatic electrochemical glucose sensor, single-frequency EIS method was utilized. The oscillation amplitude was fixed in the frequency range of 10 kHz to 0.1 Hz. The optimized working potential (DC potential) was − 0.30 V vs Ag/AgCl. EIS sensing of glucose was initially studied at GO_x_/poly (p-PDA)/CNTs/Si. The obtained Nyquist plot after circuit fitting and corresponding calibration plot in the concentration range of 0 to 1.2 mM are represented in Fig. S3A and S3B, respectively in the supplementary information. Further, impedimetric sensing of glucose was performed on GO_x_/poly (p-PDA)/CNTs/Au MEA (c.f. Figure 8B and C). The frequency for analytical measurements and the ideal parameters of the complex impedance (module, phase, real, or imaginary impedance) were considered to determine the accuracy based on the larger slope and correlation coefficient as a function of glucose content. The obtained calibration plot for glucose sensing at GO_x_/poly (p-PDA)/CNTs/Au MEA displays the imaginary impedance values (Z_im_) at 0.1 Hz to 10 kHz with the best linear response for the concentration of glucose in the range of 0.2 to 27.5 µM (c.f. Figure 8D). The variation of R_ct_ with a concentration of glucose can be expressed using the relation [23];

$${\mathrm{R}}_{\mathrm{ct}}=\frac{RT}{{n}^{2}{\mathrm{F}}^{2}A{k}_{ct}{C}_{0}}$$

where *n* represents the number of electrons transferred per molecule of the redox couple, *A* is the geometric surface area of the electrode, i.e., 1 × 10^−6^ cm^2^ (dimension = 10 µm × 10 µm), *k*_ct_ is the charge transfer rate constant, *C*_0_ is the bulk concentration of analyte while *R*, *F*, and *T* have their usual meanings and their values were 8.314 J.mol^−1^.K^−1^, 96,480 C.mol^−1^, and 298 K, respectively. Keeping other parameters fixed, the determination of dual linear relation as 1/R_ct_ = k[glucose] can be obtained, where k denotes all constants. This indicates a progressive lowering in the value of the charge transfer resistances upon the addition of glucose to the test solution. The significant lowering in charge transfer resistance (R_ct_) in the EIS measurements with consecutive addition of glucose may be ascribed to the enzymatic activity. The enzymatic reaction enables an electron transfer between the substrate molecule and an electrode. The reduction in the R_ct_ values depends on the extent of the applied DC potential. At low analyte concentrations, variations onto the surface of the sensor are seen which is associated with the capacitive signal of the imaginary impedance. The occurrence of two linear concentration ranges might be attributed to the increase in hindrance for the charge transfer occurring at the fabricated electrode–electrolyte interface with an increase in glucose concentration. Further, the loss in linear response was seen above 27.5 µM glucose concentrations due to the saturation of active sites of the immobilized enzyme and significant accumulation of oxidized intermediates at the surface of the electrode [24]. The calibration equations for the dual range for glucose detection are:

$$Y = 168.029[glucose] + 7.328 {R }^{2} = 0.9863 \mathrm{ For conc}.\mathrm{ range } 0.2\mathrm{ to }2.75 \upmu M$$

$$Y = 23.312[glucose] + 0.001 {R }^{2}= 0.9963 \mathrm{ For conc}.\mathrm{ range }2.75\mathrm{ to }27.5 \upmu M$$

The obtained results indicate the occurrence of dual concentration ranges from 0.2 to 2.75 µM and 2.75 to 27.5 µM. The sensitivities for the respective ranges were found to be 168.02 kΩ^−1^ M^−1^ and 23.31 kΩ^−1^ M^−1^. Again, the lowering in sensitivity of the sensor at concentrations higher than 2.75 µM was attributed to the increased hindrance for the transfer of charge between the electrode and electrolyte interface with an increase in glucose concentration. Further, the limit of detection (LOD) was calculated using the expression, LOD = *kS*_b_/*S* [25], where *S* represents the sensitivity of the method (calculated from the slope of calibration plot), *S*_b_ is the standard deviation of the blank measurement, and *k* is the statistical constant (a value of *k* = 3 is highly suggested by IUPAC, based on the confidence interval). The detection limit was observed to be 0.2 ± 0.0014 µM in aqueous PBS for three consecutive measurements.

Thus, the use of CNTs/Au MEA produced a significant increase in the current owing to the enhanced surface area due to CNTs. Further, the entrapment of the GO_x_ enzyme in the polymer matrix of p-PDA prevents the leaching of the enzyme. Therefore, GO_x_/poly (p-PDA)/CNTs/Au MEA-based glucose biosensor have shown improved conductivity, stability, specificity, and electrocatalytic properties owing to the synergistic effects of CNTs/Au MEA, GO_x_, and poly (p-PDA). It may be concluded that the fabricated GO_x_/poly (p-PDA)/CNTs/Au MEA-based biosensor possesses satisfactory linear ranges for qualitative and quantitative glucose determination in real serum samples and thus, impedimetric glucose biosensor can be fabricated successfully. The comparison of various analytical performances of the proposed electrochemical sensing platform with several other reported works [26] are summarized in Table 1. The proposed sensor has shown an LOD of 0.2 ± 0.0014 µM and it is lowest than that of GCE-RFG-RGO-GOD-CS [25], GOx-GQD/CCE [27], GCE/PG/NF/FC/GOD [30], PNE/GOD/AuNPs@PNE/Au [28], PNE/GOD/AuNPs@PNE/Au (with BQ) [28], NPPt/GO/GOx/Nafion [29], Graphene-CdS-GOD/GCE [31], and GOx/AuNP/PANI/rGO/NH_2_–MWCNTs [32], while, it is comparable to that of PDA/GOD/GN/Au [26] for glucose detection. Moreover, GO_x_/poly (p-PDA)/CNTs/Au MEA has shown a wide linear calibration range with unparalleled sensitivity. Hence, the results obtained for the developed glucose biosensor were found to be on par/better than the various reported works in terms of the detection range, LOD and sensitivity.

**Table 1:** Comparison of different enzymatic glucose sensors based on their electrochemical performances

Electrode	Method (measurement time)	Linear range (mM)	LOD (µM)	Sensitivity (µAmM^−1^ cm^−2^)	References
GCE-RFG-RGO-GOD-CS	Amperometric	-	79.65	46.71	[25]
NPPt/GO/GOx/Nafion	Amperometric (3 s)	0.1–20.0	13	11.64	[29]
Graphene-CdS-GOD/GCE	Impedimetric	2.0–16.0	700	-	[31]
PDA/GOD/GN/Au	Amperometric (< 4 s)	0.001–4.7	0.1	28.4	[26]
GOx-GQD/CCE	Amperometric (> 3 s)	0.005–1.270	1.73	85	[27]
GCE/PG/NF/FC/GOD	Impedimetric	1–15	21	10.6 kΩ.mM^−1^	[30]
PNE/GOD/AuNPs@PNE/Au	Amperometric (< 3 s)	0.003–3.43	1.34	35.4	[28]
PNE/GOD/AuNPs@PNE/Au (with BQ)	Amperometric (< 3 s)	0.003–8.044	1.76	59.17	[28]
GOx/AuNP/PANI/rGO/NH_2_–MWCNTs	Amperometric	1.0–10.0	64	246	[32]
GOx/poly (p-PDA)/CNTs/Au MEA	Impedimetric (15 min)	0.0002–0.027	0.2	168.03 kΩ^−1^ M^−1^	This work

We cannot predict the exact estimated value of the proposed array of 64 microelectrodes and after modification to generate a glucose biosensor. However, it may cost approximately US $80–100 for the fabrication of one GOx/poly (p-PDA)/CNTs/Au MEA. In addition, as the prepared MEAs have 64 microelectrodes and we are using a single microelectrode at a time for glucose sensing and when glucose analysis is performed on large scale by utilizing this fabricated sensor, the analysis of one glucose sample may cost less than US $1 only. There are no major limitations of the work presented here in terms of sensor development and its application in the electrochemical determination of glucose. However, there was one problem related to the connectivity of one specific microelectrode at a time while electrochemical measurement. Since the connecting pads of each microelectrode of the array are closely spaced, there was difficulty in connecting any microelectrode to the electrochemical system and hence, multi-analyte detection by using each microelectrode is slightly difficult. However, this issue may be resolved by developing a prototype that can be used for making a connection with any particular microelectrode at a time, and thus, each microelectrode can be used separately for the detection of a specific analyte.

### Sensor performance

#### Reproducibility study

Reproducibility is one of the important factors that signify the feasibility and potential applicability of the developed electrochemical sensor. For this, five different microelectrodes (out of 64) within the same CNTs/Au MEA were chosen and modified under similar conditions. Then, the reproducibility of the developed glucose biosensor was investigated through EIS measurements (c.f. Fig. S4) on each modified microelectrodes in PBS (pH 6.5) containing 22 µM glucose. The relative standard deviation (RSD) of 1.28% was found at five different modified microelectrodes, which confirms that the GO_x_/poly (p-PDA)/CNTs/Au MEA possesses excellent reproducibility.

#### Stability and reusability of prepared electrode

The three consecutive measurements at one microelelectrode in the presence of PBS (pH 6.5) containing 10 µM glucose concentration have shown < 4% RSD. Moreover, the stability of the GO_x_/poly (p-PDA)/CNTs/Au MEA-based biosensor was investigated for a period of 15 days by keeping the fabricated electrode at 4 °C in the refrigerator. The EIS measurements were performed for measuring the response of 10 µM glucose concentration in 0.1 M PBS (pH 6.5) at one microelectrode of GO_x_/poly (p-PDA)/CNTs/Au MEA. It was observed that the developed sensor has shown 1.19% and 3.13% reduction in R_ct_ values on 7th and 15th day, respectively with respect to the initial value (c.f. Fig. S5). This shows that the activity of the GO_x_/poly (p-PDA)/CNTs/Au MEA-based biosensor is maintained even after 15 days. The lifetime of the fabricated CNTs/Au MEA was found to be more than 2 years; further, it can be kept at room temperature in dust free environment if, of no use with neutral loss in response.

Further, the immobilized enzymes are robust to environmental changes and retain their catalytic activity compared to free enzymes. Thus, the reusability of the prepared biosensor was also investigated by measuring the response of 10 µM glucose concentration in 0.1 M PBS (pH 6.5) on one microelectrode (out of 64) of GO_x_/poly (p-PDA)/CNTs/Au MEA for number of reactions through EIS study. The fabricated sensing platform was rinsed several times with 0.1 M PBS (pH 6.5) and dried after each measurement. The reusability study as represented in Fig. S6 suggests that poly (p-PDA) immobilized GO_x_ exhibited a variation in Rct value after each reaction and a reduction of 5.97% in R_ct_ value was observed with respect to initial value after 10 continuous reactions. This might be attributed to the loss in activity of immobilized GO_x_ enzyme due to the frequent interaction of substrate within the same active site of the enzyme. Therefore, the proposed sensor can be used repeatedly for glucose detection for 10 continuous reactions with a storage stability of 15 days and it reduces the cost of production.

#### Interference studies

Interference testing is a good approach for determining the selectivity of a developed biosensor based on GO_x_/poly (p-PDA)/CNTs/Au MEA system for glucose detection. In the presence of possible interferents, we observed the behavior of the fabricated sensor system. The EIS study was performed after adding some common interfering substances such as ascorbic acid, citric acid, cysteine, uric acid, urea, dopamine, cholesterol, and paracetamol (5 mM each) to the 0.1 M PBS (pH 6.5) containing 22 µM glucose as a fixed concentration. The results (c.f. Fig. S7) showed that there is a minimal change in the charge transfer resistance in the presence of the added interferents showing the high selectivity of the proposed glucose biosensor. Although ascorbic acid and cysteine are reducible, no influence on glucose sensing has been described in the literature. Furthermore, the designed sensor has shown high selectivity for glucose in the presence of electroactive substances such as dopamine, uric acid, and paracetamol that might be due to the specificity of GO_x_ and glucose.

#### Glucose determination in human blood serum sample

The determination of glucose concentration in a real sample was achieved using the fabricated GO_x_/poly (p-PDA)/CNTs/Au MEA-based biosensor through EIS measurements at an applied potential of − 0.30 V vs Ag/AgCl. For the preparation of a real sample, 250 folds dilution of 8 µL human serum with 0.1 M PBS (pH 6.5) was done according to the guidelines on bioanalytical method validation so that the concentration of glucose in the given sample should lie in the validated range of analysis [33]. However, the dilution ratio should be at the smallest so that accuracy and precision of the analysis should be maintained. The prepared sample was directly taken in the electrochemical cell and then the recovery analysis of glucose was carried out impedimetrically. The standard addition method has the ability to overcome the samples matrix interference and it can also determine the low concentration of the analyte. Hence, the standard addition method was employed for analysis in the prepared real samples. Each sample was analyzed for three times under identical conditions. Finally, the recovery in terms of percentage was calculated by applying the following formula [34].

$$Recovery\;(\%)=\frac{C_{\mathrm{spiked}\;\mathrm{sample}}-C_{\mathrm{unspiked}\;\mathrm{sample}}}{C_{\mathrm{added}}}\mathrm X100$$

Where $C_{\mathrm{spiked}\;\mathrm{sample}}$ = concentration of glucose in spiked real sample,

$C_{\mathrm{unspiked}\;\mathrm{sample}}$ = concentration of glucose in unspiked real sample.

${C}_{\mathrm{added}}$ = concentration of glucose added.

The results represented in Table 2 reveal the recovery between 98.75 and 105% for the glucose determination at the developed sensing platform which shows the presence of negligible interference by the blood serum matrix. The obtained results were compared through an alternative interference free method, i.e., *the* HPLC method and it was observed that results of electrochemical analysis have shown consistency with those obtained by the HPLC method with acceptable recoveries. Therefore, the developed glucose biosensor was effectively used for glucose concentration analysis in the diluted human blood serum sample.

**Table 2:** Determination of Glucose in human serum samples

Samples	Added (μM)	Expected (μM)		Found (μM)		Recovery (%)	
		HPLC	This method	HPLC	This method	HPLC	This method
1	0.0	-	-	18.5	18.4	-	-
2	0.2	18.70	18.60	18.72	18.61	110.00	105.00
3	2.0	20.50	20.40	20.46	20.45	98.00	102.50
4	8.0	26.50	26.40	26.80	26.30	103.75	98.75

## Conclusions

The present study discusses the preparation of photolithographically patterned MEAs on a glass substrate for the fabrication of a significantly sensitive electrochemical enzymatic glucose biosensor. The developed GO_x_/poly (p-PDA)/CNTs/Au MEA-based biosensor was used for measuring the glucose concentration through the EIS technique based on enzymatic glucose oxidation. Leaching of the GO_x_ enzyme was prohibited by immobilizing the enzyme in a polymer matrix. A wide linear detection range was covered between 0.2 and 27.5 µM by observing the change in R_ct_ value with the addition of several glucose concentrations at a fixed DC potential. Also, the developed biosensor possesses acceptable characteristics such as high detection sensitivity, exceptional reproducibility, and excellent shelf life. These results are very encouraging and it may be concluded that the fabricated GO_x_/poly (p-PDA)/CNTs/Au MEA-based glucose biosensor is scientifically valid, practically operative, and shows the promising application in the glucose concentration determination in real samples. The CNTs/Au MEA has an array of 64 microelectrodes and one analyte can be detected on one microelectrode of the fabricated array, and hence, it might be possible for our research group to develop an electrochemical sensor for the simultaneous detection of multiple analytes in the future.

## Supplementary information

Below is the link to the electronic supplementary material.

Supplementary file1 (DOCX 2.25 MB)

## Data Availability

Most data generated or analysed during this study are included in this published article and its supplementary information file.
