# Supplementary material for: Electrochemical biosensors based on in situ grown carbon nanotubes on gold microelectrode array fabricated on glass substrate for glucose determination
Source: Mikrochim Acta. 2023 Jan 16;190(2):55. doi: 10.1007/s00604-022-05626-6 (PMC9842592; doi:10.1007/s00604-022-05626-6)
Supplement: Supplementary file 1 — Supplementary file1 (DOCX 2.25 MB) [file 604_2022_5626_MOESM1_ESM.docx]

**Supplementary Information for:**

**Electrochemical biosensors based on in situ grown carbon nanotubes on gold microelectrode array fabricated on glass substrate for glucose determination**

Ankit Kumar Singh^a^, Nandita Jaiswal^a^, Ida Tiwari^a*^, Muhammad Ahmad^b^ and S. Ravi P. Silva^b*^

^a^Department of Chemistry (Centre of Advanced Study), Institute of Science,

Banaras Hindu University, Varanasi-221005, India

^b^Advanced Technology Institute, University of Surrey, Guildford, Surrey, GU2 7XH, UK

**^*^*Corresponding authors***

**Tel:** +91-9415813020; Fax: +91-542-2368174; +44 (0)1483 689825

**E-mail:** [idatiwari@bhu.ac.in](mailto:idatiwari@bhu.ac.in), [s.silva@surrey.ac.uk](mailto:s.silva@surrey.ac.uk)


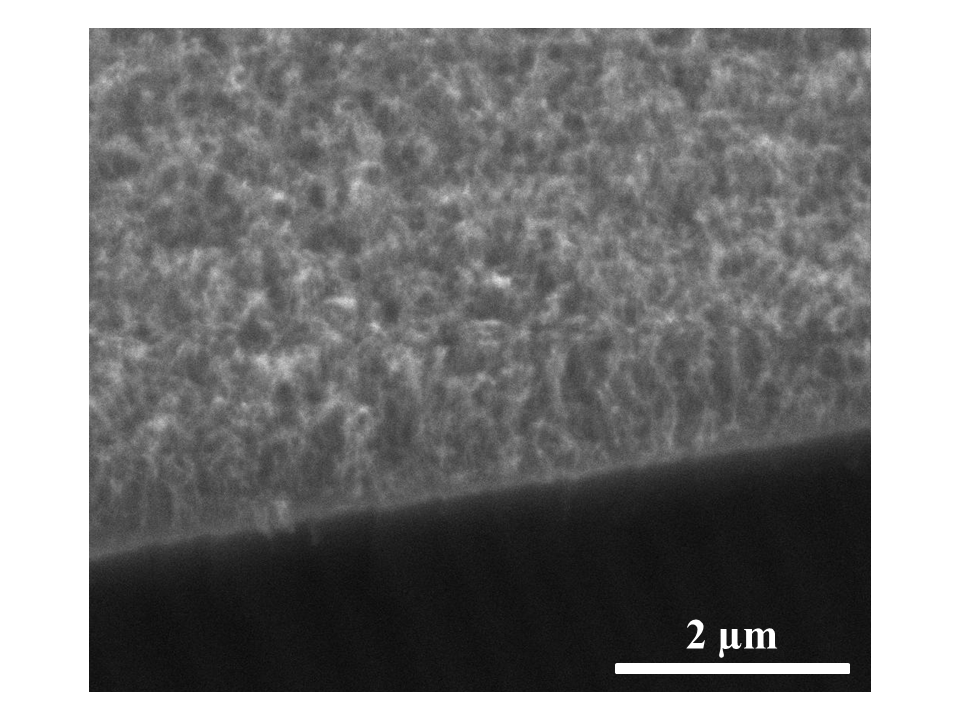


**Fig. S1** SEM image of CNTs grown on silicon wafer.


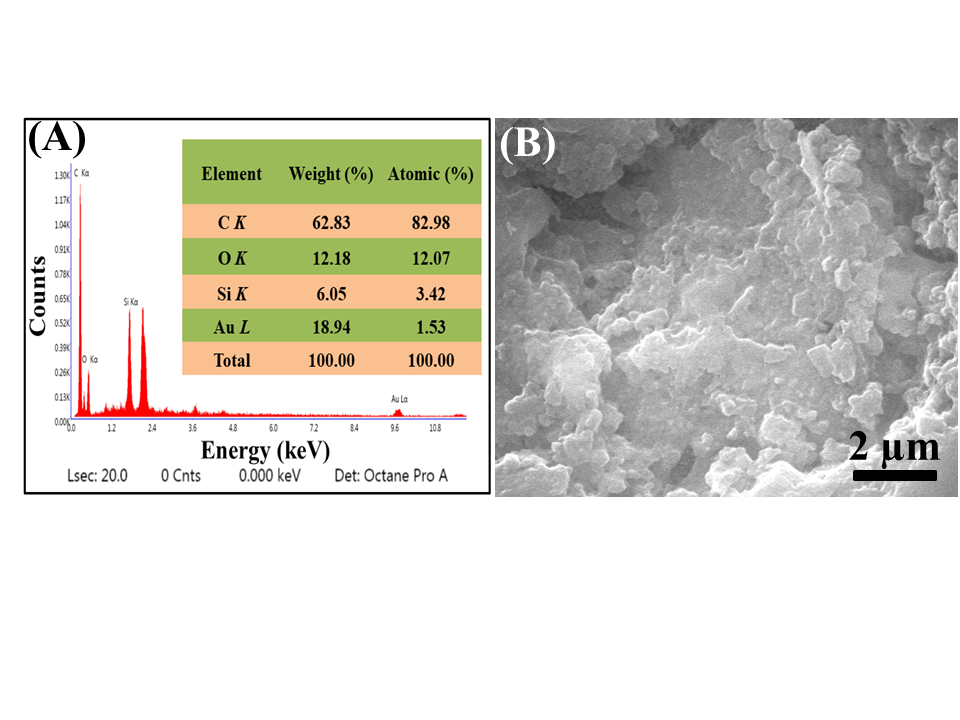


**Fig. S2** (A) EDX analysis of CNTs/Au MEA. SEM image of GO_x_/poly (p-PDA)/CNTs/Au microelectrode.


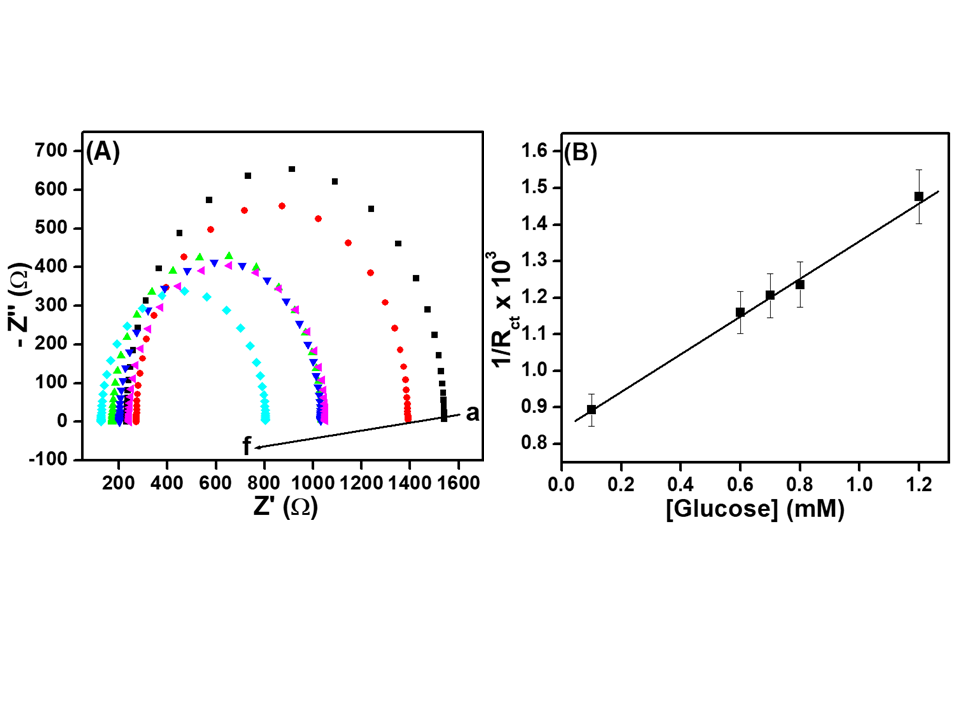


**Fig. S3** **(A)** EIS response of glucose on GO_x_/poly (p-PDA)/CNTs/Si. **(B)** Calibration plot for glucose addition on GO_x_/poly (p-PDA)/CNTs/Si in concentration range of 0.1 to 1.2 mM in PBS of pH 6.5.





**Fig. S4** EIS response of 22 µM glucose on five different microelectrodes (out of 64) of GO_x_/poly (p-PDA)/CNTs/Au MEA in PBS of pH 6.5.





**Fig. S5** Stability of GO_x_/poly (p-PDA)/CNTs/Au MEA towards determination of 10 µM glucose in 0.1 M PBS of pH 6.5.





**Fig. S6** Reusability study towards determination of 10 µM glucose in 0.1 M PBS (pH 6.5) at one microelectrode of GO_x_/poly (p-PDA)/CNTs/Au MEA.





**Fig. S7** Interference study of the developed sensor system towards 22 µM glucose detection in the presence of several interferents in 0.1 M PBS of pH 6.5.
